# Supplementary material for: Morphology-Tailored Gold Nanoraspberries Based on Seed-Mediated Space-Confined Self-Assembly
Source: Nanomaterials (Basel). 2019 Aug 27;9(9):1202. doi: 10.3390/nano9091202 (PMC6780137; doi:10.3390/nano9091202)
Supplement: Supplementary file 1 [file nanomaterials-09-01202-s001.pdf]

Supporting Information:

Morphology-Tailored Gold Nanoraspberries Based on Seed-Mediated Space-Confined Self-Assembly

Yan Yu [1], Yujun Xie [1], Pan Zeng [1], Dai Zhang [1], Rongqing Liang [1], Wenxing Wang [2,*], Qiongrong Ou [1,*] and Shuyu Zhang [1,*]

[1] Department of Light Sources and Illuminating Engineering, Engineering Research Center of Advanced Lighting Technology (MoE), and Academy for Engineering and Technology, Fudan University, Shanghai 200433, China

[2] Department of Chemistry and Laboratory of Advanced Materials, Fudan University, Shanghai 200433, China

* Correspondence: wangwenxing@fudan.edu.cn (W.W.); qrou@fudan.edu.cn (Q.O.); zhangshuyu@fudan.edu.cn (S.Z.)

1. Scatter plots for size distribution of AuNC and AuNRbs during different growth stages.

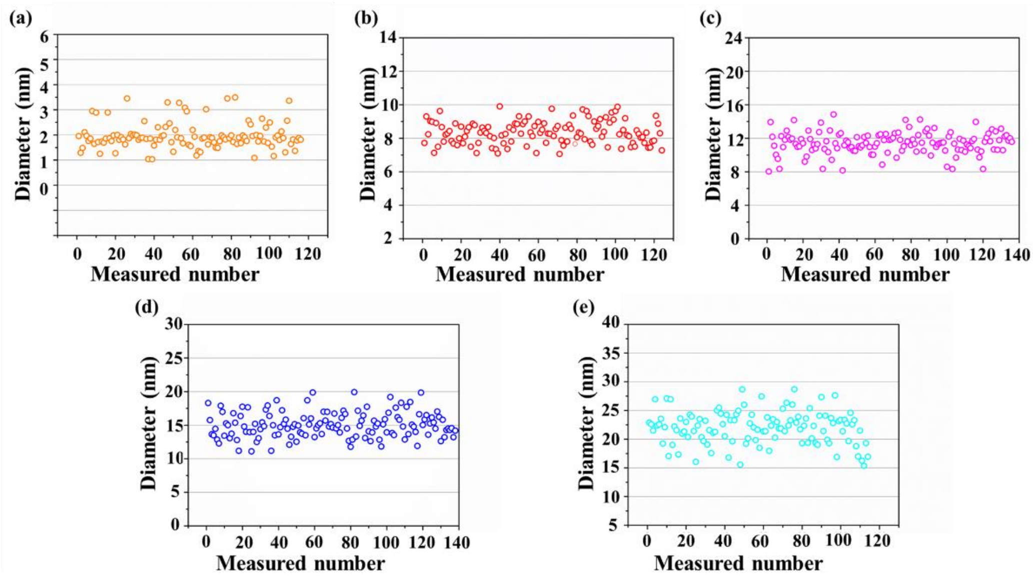

Figure S1 The scatter plots of size distribution of AuNC and AuNRbs during different growth stages corresponding to Figure 2(a)~(e).

2. TEM images for morphology stability characterization.

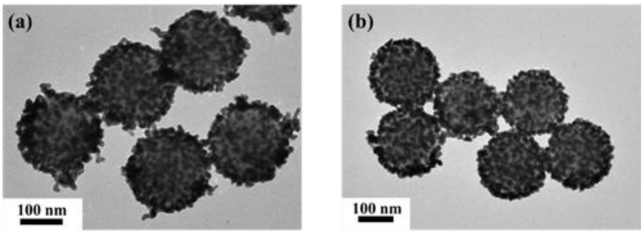

Figure S2 The TEM images of AuNRb-15 (a) when freshly prepared and (b) after a storage of four months.

3. Growth mechanism of gold nanoparticles on S-SiO_2 templates.

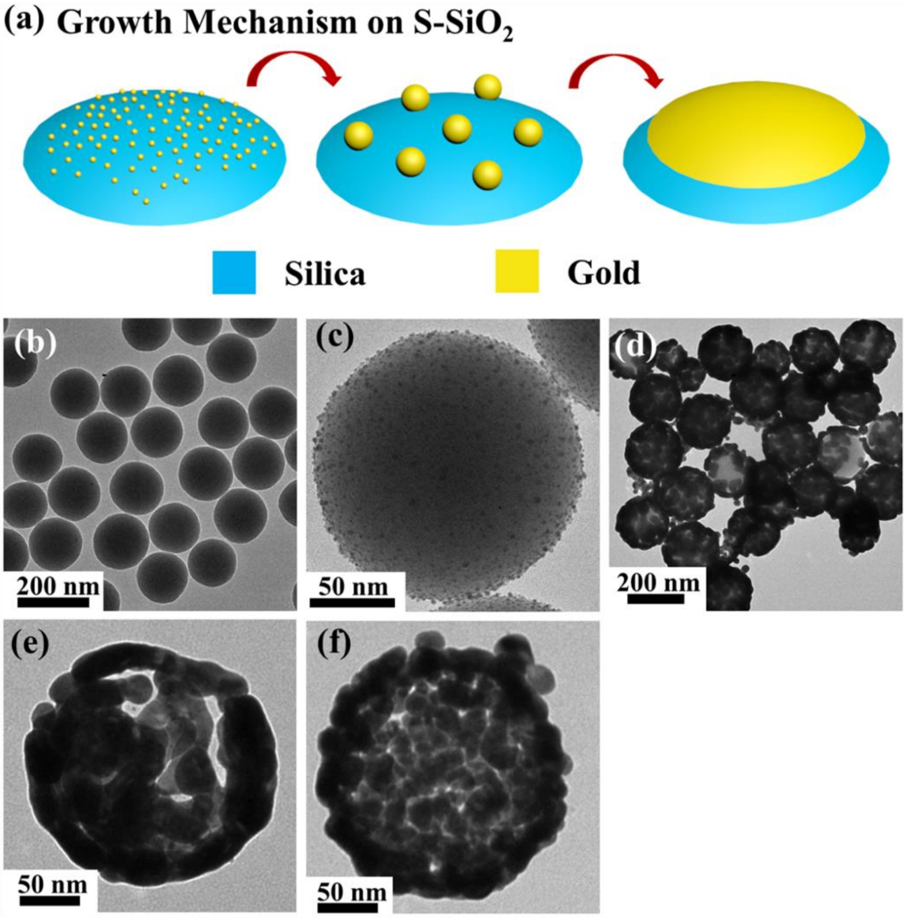

Figure S3 (a) A schematic diagram illustrating the aggregative growth of gold nanoparticles on the surface of smooth silica (S-SiO_2) nanoparticles. TEM images of (b) S-SiO_2 nanoparticles; (c) an S-SiO_2 nanoparticle attached by gold nanoseeds; and (d) S-SiO_2 template-based gold nanoshell. TEM images of gold products synthesized by (e) S-SiO_2 and (f) V-SiO_2 nanoparticles after the removal of templates.

4. A TEM image of AuNP-20.

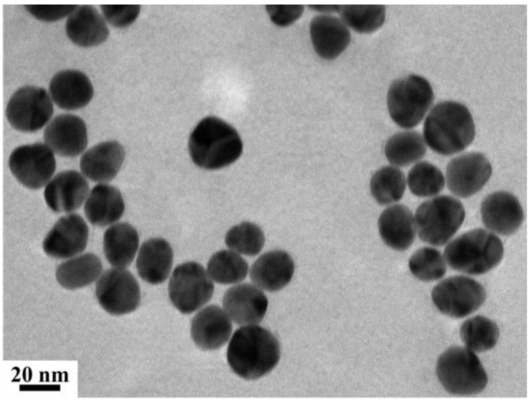

Figure S4 A TEM image of AuNP-20 prepared by sodium citrate reduction.

5. Additional scattering and absorption peaks.

In addition to the red-shifted scattering peak at the wavelength of 760 nm, a new scattering peak at the wavelength of 565 nm appears, provided that the interparticle distance is kept at 4 nm and the nanoparticle size is 16 nm in diameter. The y-component of the time-averaged power flow of relative field at the wavelength of 760 nm and 565 nm is plotted in Figure S3a and S3b, respectively. As shown in Figure S3b, the new peak supports a high-order plasmonic resonance. This phenomenon has also been found when the diameter of the nanoparticle is kept at 10 nm and the interparticle distance is set at 0.9 nm. The y-component of the time-averaged power flow of relative field at the wavelength of 730 nm and 615 nm is plotted in Figure S3c and S3d, respectively. Moreover, the interparticle distance has a strong influence on the absorption peak. When the diameter of the nanoparticle is kept at 10 nm and the interparticle distance is reduced from 5.2 nm to 0.9 nm, the absorption peak shows a distinct red-shift from 520 nm to 600 nm which is caused by the circumferential absorption enhancement due to the interparticle resonance. Figure S3e and S3f shows the total power dissipation density when the interparticle distance is 5.2 nm and 0.9 nm, respectively. In Figure S3e, the power dissipates fairly uniformly over the entire volume of each gold nanoparticle. On the contrary, in Figure S3f, the power dissipation reaches maximum values at the outskirts of the gold nanoparticles.

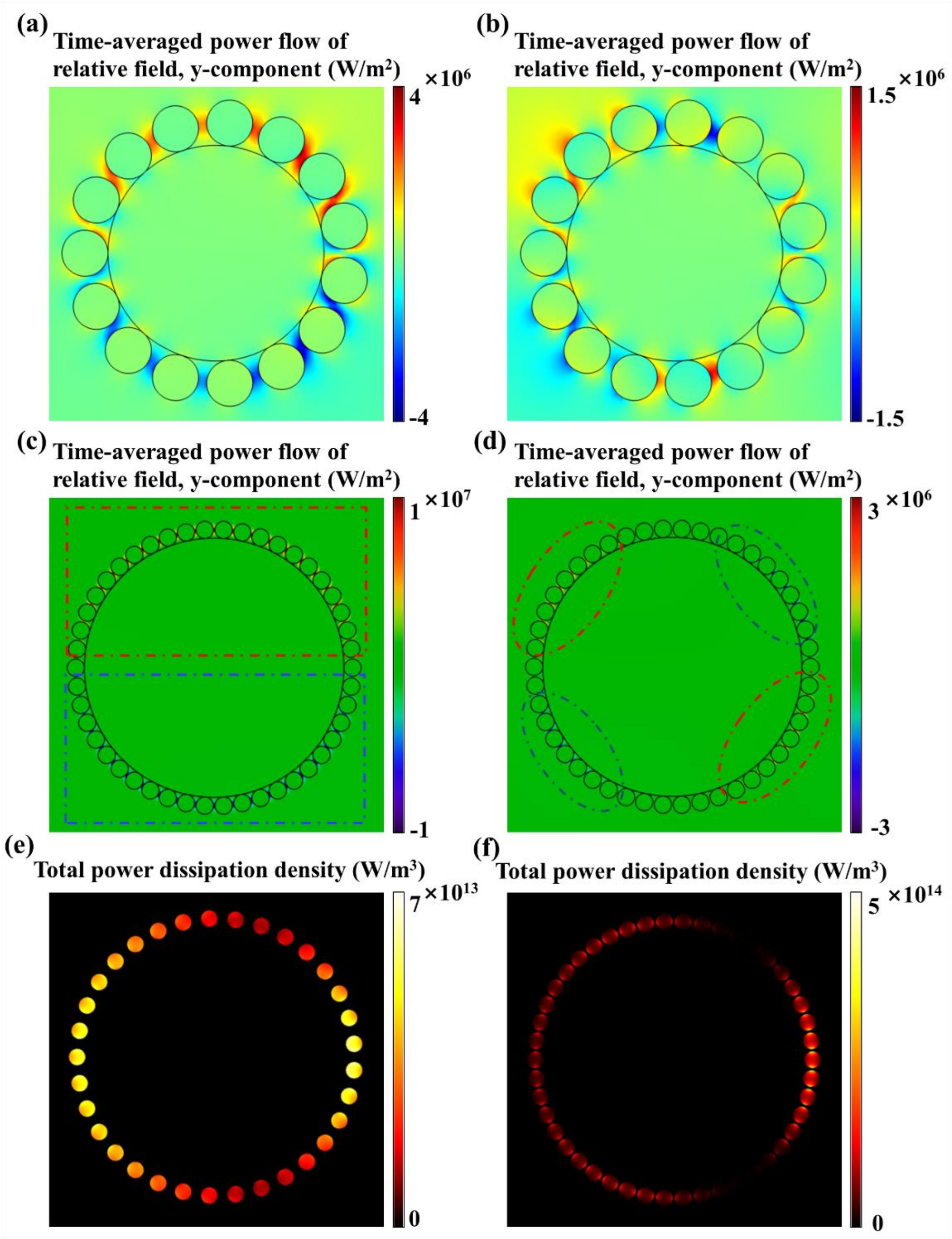

Figure S5 The y-component of the time-averaged power flow of relative field at the wavelength of (a) 760 nm and (b) 565 nm when the interparticle distance is 4 nm and the nanoparticle size is 16 nm in diameter. The y-component of the time-averaged power flow of relative field at the wavelength of (c) 730 nm and (d) 615 nm when the interparticle distance is 0.9 nm and the nanoparticle size is 10 nm in diameter. The total power dissipation density when (e) the interparticle distance is 5.2 nm at the wavelength of 520 nm, and when (f) the interparticle distance is 0.9 nm at the wavelength of 600 nm.
